# Cultural adaptation, the 3-month efficacy of visual art training on observational and diagnostic skills among nursing students, and satisfaction among students and staff- a mixed method study

**DOI:** 10.1186/s12912-021-00646-8

**Published:** 2021-07-06

**Authors:** Jia Guo, Qinyi Zhong, Ying Tang, Jiaxin Luo, Hongjuan Wang, Xiaofen Qin, Xiuhua Wang, James Allen Wiley

**Affiliations:** 1 Xiangya School of Nursing, Central South University, NO.172 Tongzipo Road Yuelu District, Changsha, 410013 Hunan Province China; 2 School of Architecture and Art, Central South University, Changsha, Hunan Province China; 3 Hainan General Hospital, Haikou, Hainan Province China; 4 Department of Family and Community Medicine, University of California, San Francisco, USA

**Keywords:** Culture, Art, Observation, Diagnosis, Clinical competence, Nursing education, Satisfaction

## Abstract

**Background:**

Visual art training is a student-led approach using Western art pieces as the main teaching resources. It has been developed and applied in nursing and medical education in the United States. This study aimed to adapt visual art training to Chinese cultural context, then to compare the efficacy of the culturally-tailored visual art training versus traditional education on observational and diagnostic skills at 3-month follow-up among Chinese nursing students in master program.

**Methods:**

This study included Phase 1 (cultural adaptation) and Phase 2 (3-month efficacy evaluation). It was conducted from June to September, 2019. In Phase 1, cultural barriers were identified and cultural adaptation strategy were made based on two focus group interviews. Phase 2 was a randomized controlled trial in a local museum. A total of 106 first-year nursing students in master program were randomized to the intervention group or the control group. Both groups received traditional education. In addition, intervention group received a visual art training (including a field-guided museum visit with observation and debriefing of Chinese oil paintings and clinical images, four teaching hours). Data were collected for both groups at baseline and 3-month follow-up on the observational and diagnostic skills measured by clinical image tests. Learning satisfaction with the visual art training was investigated among 53 intervention students and teaching satisfaction was done in 10 staff members by self-administered questionnaires.

**Results:**

In phase 1, we adapted a culturally-tailored visual art training for nursing students in China. Observational skills of the intervention group increased significantly compared with the control group 3 months after the training (*p* < .001). A trend towards the improvement of diagnostic skills was indicated with increment of 2.92 points of the intervention group vs. 0.39 of the control group (*p* > .05). In general, all participants and staff were satisfied with the visual art training, especially the selected Chinese oil paintings and the student-led teaching process, but 34% (*n* = 18) were not satisfied with the long distance from the museum.

**Conclusions:**

A culturally-tailored visual art training with great acceptability and feasibility was implemented in China. It had a sustained positive effect on improving the observational skills of Chinese nursing students. This study can be used for a reference to introduce visual art training to nursing students or nurses from other cultures.

**Trial registration:**

Retrospectively registered in Chinese Clinical Trial Registry (ChiCTR2000037956) on 4th September, 2020.

## Background

Nursing diagnosis making begins with observations, then gather information derived from observations, analyze information, and end with conclusions [1]. There is general agreement that accurate observations are key components of nursing diagnosis-making by providing careful, unbiased visual information [2]. For example, Chang et al. found that medical students who are able to observe key information in clinical cases are more likely to arrive at accurate diagnoses [3].

An observation is the process of noting and identifying something or someone to gain information, which involves more than just a cursory glance at an object or a patient [4]. Observational skills teach us about objects, events, attitudes, and experiences by our visual sense [5]. The development of observational skills is essential for nursing students, as it directs them to move away from simply making assumptions to critically diagnosing patients and examining situations [6].

Many studies show inadequacy in observational skills among most nurses and nursing students in China and elsewhere, which may be owing to lack of explicit teaching of observation [7, 8]. Unlike memorization of rules, practices, and experiential and theoretical knowledge, observation is a unique skill that cannot be learned formally through books or lectures [9]. However, courses in nursing schools tend to emphasize the identification of memorized clinical signs rather than formally teaching students how to observe [10]. It is clear that better teaching methods for observational skills need to be introduced in nursing schools.

There are many specialized teaching methods for observational skills with the common goal to deliver experiences that modify the way learners process visual information to foster the development of observational skills. These include: 1) visual art training, which engages students in visual observation to find meaning in works of art under the guidance of educators by three questions: “What do you see?”, “What makes you say that?”, and “What else do you see?” [11]; 2) experiential learning activities in which students assume the role of the patient in simulated clinical scenarios [12]; 3) narrative interventions, which use literary and theatrical media to immerse students within the contexts of illness and care [13]; and 4) observation skills workshops, which use lectures or video recordings to highlight the correct way to observe [14]. Among these methods, visual art training as a cost-effective approach is developed to combine clinical scenarios, and is highly recommended for nursing educators by the Carnegie Foundation [15, 16].

Visual art training is a student-led pedagogical method with Western art pieces used as the main teaching resources. The goal of this pedagogical method is to impart experience concerning how to observe patients and situations in clinical scenarios by viewing and critiquing art pieces exhibited in a museum [17]. Pellico and her colleagues found that nursing students observed more symptoms and identified more objective clinical findings right after a half-day visual art training [9]. Previous studies with medical students showed that three-week visual art training can improve observational skills in the short term, appreciation of multiple perspectives, and their relationship to clinical practice [18]. According to a narrative review, these trainings achieved excellent effectiveness, but almost all of results were reported right after the training [19]. Whether or not the skill retention can be achieved by visual art training needs additional assessment.

The visual art training for nursing or medical students is rarely reported in other countries out of the United States. In 2018, our research team introduced the visual art training which has been conducted at Yale University since 2009 to mainland China [8]. A total of 29 Chinese nursing students in master program interpreted six Western art pieces in a simulated art museum, and debriefed four clinical images using the same procedure for art pieces in this training. However, we found student involvement was low, especially when each student was encouraged to interpret one Western art painting based on their personal experience. To address low student involvement, we explored reasons from a cultural difference perspective and designed a new randomized controlled trial that modifies the original visual art training to make it culturally more relevant to nursing students in China.

This study aimed to a) adapt visual art training to Chinese cultural context; b) compare the 3-month efficacy of this culturally-tailored visual art training versus traditional education on the observational (primary outcome) and diagnostic skills (secondary outcome) among Chinese nursing students in master program; and c) investigate the learning satisfaction with this culturally-tailored visual art training among participants and the teaching satisfaction among staff.

## Methods

### Study design

This study included two consecutive phases: 1) cultural adaptation of visual art training; 2) the 3-month efficacy evaluation with a randomized clinical trial. Approval for adapting the visual art training to Chinese culture was authorized by the developer of the original training, Dr. Pellico Linda [9]. The study was approved by the ethical review board of our university (No.2017037) and registered on the Chinese Clinical Trial Registry (ChiCTR) (No. ChiCTR 2,000,037,956).

### Phase 1: cultural adaptation process of the visual art training

Based on Barrera’s heuristic framework concerning cultural adaptation of interventions, this phase included two consecutive steps: information gathering and adaptation design [20].

#### Step 1: information gathering (identification of cultural barriers)

In this step, two focus group interviews were simultaneously conducted among seven student participants and eight staff members a week after completion of the pilot study. The goal of the two interviews was to identify the potential cultural barriers in the pilot study to introduce the visual art training to Chinese cultural context. The staff members were interviewed separately from the student participants to guarantee that barriers could be fully proposed from both of learning and teaching perspective.

All the student participants were female with the average age of 25.4 (SD 2.8) years old. Most of them (*n* = 5) had not received some formal art education before recruited. In addition, the staff members who conducted the teaching process (refer to museum guides) or who supervised the process (refer to experts) at the simulated museum were also invited to the interview. These staff members included four museum guides who were at least bachelors of arts and familiar with art pieces used in pilot study well, and four experts (one clinical professor, two nursing professor, and one art professors) who obtained doctoral degree of nursing or arts and were systematically trained the teaching approach of visual art training by Pellico and her colleagues in Yale University. All of the staff members were over 35 years old with at least 5 years working experience or teaching experience.

These student participants and staff members were asked to share their learning experience (or teaching experience), what they liked and disliked, and any comments or questions that came to mind as they reviewed the training. The emphasis of the interview was on a constructive critique, rather than approval. Focus group interviews were audio recorded and transcribed verbatim. NVIVO (Version 10, QSR International) was used to organize, code, and analyze the data [21].

The student participants and staff members enthusiastically supported for the application of the visual art training in Chinese nursing education and their feedback regarding areas for improvement was elicited. The student participants thought Western art pieces used in the training were the main barrier to get them involved. For example, a student said, “*I think it is interesting and I want to take an active part, but it was hard for me to initiatively interpret these Western art pieces without cues from the teacher, because most Western art pieces had intricate historical backgrounds and mysterious religious color for me*”. In addition, staff members suggested student involvement was the core component of the training and needed to be improved. For example, one staff member said, “*There was an unconscious shift from student- to teacher-led training during the implementation process, which was not what we expected"*. “*This might be due to shy nature of Chinese students and obscure Western art pieces. We should work on this*”. Based on the systematically assessment and unanimous agreement of students and staff, Western art pieces and low student involvement were identified as major cultural barriers and needed to be addressed. The detailed comments from students, experts in art, and experts in nursing were displayed in the Appendix.

#### Step 2: adaptation design (cultural adaptation strategy making)

After the cultural barriers were determined, an expert workshop was followed immediately among staff members. A series of consensus-based cultural adaptation strategies were put forward to designed a culturally-tailored visual art training for Chinese students.

#### The selection of Chinese oil paintings

With the development of society and culture, there have been a few art museums in provincial capitals (including our research site) in China. A local art museum was chosen for field visit in the training with free access to a collection of Chinese oil paintings and covering an area of 15.3 hectares. Compared with in a simulated museum, there are more paintings and better learning atmosphere to inspire students get more involved in the training in a real art museum.

All Western art pieces used in the previous pilot study were replaced with Chinese oil paintings exhibited in this museum. The staff members reformulated the inclusion criteria of art pieces: having obvious Chinese characteristics, rich visual detail, and potential for ambiguity of interpretation without predetermined correct answers. The art pieces with obscure historical backgrounds, mysterious religious color and art background knowledge were excluded.

The preliminary selection of Chinese oil paintings was done by working with a local museum manager according to the inclusion and exclusion criteria of art pieces. A total of 17 Chinese oil paintings in the museum were selected by the museum manager. After that, these 17 paintings were rated (highly recommend = 4, moderately recommend = 3, slightly recommend = 2, not recommend at all = 1) separately by previously mentioned eight staff members during a field visit. The top six paintings with highest scores were selected finally (range 30 ~ 32 points).

#### Other attempts to improve student involvement

The detailed teaching plans and observation procedures of six Chinese oil paintings were developed to help improve student involvement. Compared with the original observation procedure used in the pilot study [8], we prolonged interpretation and communication process from 5 min to 10 min for each art pieces. Students were encouraged to explore various plausible interpretations about the art piece without judgement from professionals, unless their interpretations were illogical or showed idiosyncratic personal bias. “*Can anybody add some observations or interpretations?*” or “*What else do you find?*” were repeated asked to prompt students to share ideas and generate multiple interpretations via group discussions.

The museum guides were invited to lead students in this culturally-tailored visual art training. They were trained about the standard implementation procedures to guide the observations of Chinese oil paintings by reviewing the training manual developed by the research team and practicing delivery scenarios together, etc. They were also armed with intervention skills (e.g. how to encourage students to overcome their shy nature). At the end of the delivery training for museum guides, they were asked to simulate the standard procedures of visual art training one by one and the research team gave comments suggested some improvements according to their individual practices. Only receiving all delivery training, the museum guides could be qualified to deliver the visual art training.

### Phase 2: 3-month efficacy test

A randomized controlled trial was designed to test 3-month efficacy of culturally-tailored visual art training from June to September, 2019. It was conducted in a local art museum.

### Participants

Inclusion criteria were: 1) first-year nursing students educated in MSc and enrolled in 2018; 2) bachelor of nursing, which ensured that participants received similar educational curriculum before and were in the same field; 3) registered nurses; and 4) able to speak and write in Mandarin Chinese. Students were excluded if they 1) had received visual art training before (other forms of art experience were accepted); 2) had schedule conflicts that limited their ability to participate; and 3) had severe psychiatric disorders or physical disability.

Sample size was determined by G*Power according to the primary outcome-observational skills [22]. Based on the effect size d = 0.61 observed by Nease et al. in their randomized controlled study of visual art training, the required total sample size with alpha = .05 and for power = .80 is 90 [23]. Based on an estimated maximum potential attrition rate of 20% at the 3-month follow-up, a sample size of 106 was needed.

With the assistance of the administrative staff of the nursing master’s programs, eligible students were invited to enroll in our study after the purpose, benefits, and risks of the study were fully explained in the form of a PowerPoint presentation (20 min) by a member of our research team during the break time of a compulsory course. If students were interested in our study, a trained research assistant contacted them within a week, and the study was further described in detail before information registration into the study. The written informed consent was obtained from each participant.

Participants were randomly assigned 1:1 to either the intervention group (*n* = 53, receiving culturally-tailored visual art training plus traditional education) or the control group (*n* = 53, receiving traditional education) using an SPSS-generated random allocation sequence with setting 1 as the control group and 0 as the intervention group in advance by a statistician. All intervention participants signed a confidentiality agreement, which stated that the contents of the intervention should not be disclosed to the control participants. Given the nature of the intervention, it is impossible to mask the participants or staff to the group assignments during the intervention. However, group assignments were concealed during data collection and data analysis without any potential identifiers.

### Interventions

***Traditional Education*** refers to an Advanced Health Assessment Course (48 classroom hours), which is a core course for first-year nursing students in master program. It focuses on how to take comprehensive health histories, physical examination, electrocardiogram, and nursing record writing.

***Culturally-tailored Visual Art Training*** included a field-guided museum visit (3 classroom hours) and exposure to clinical images with debriefing (1 classroom hour). In the first session (field-guided museum visit), participants were assigned to nine groups (five to six participants per group) according to the registration order to observe six Chinese oil paintings in rotation in the museum, which ensured each participant in a group had opportunities to be chosen as a speaker to describe at least one painting. Each of the museum guides led one group. They used various strategies to prompt participants to view paintings interactively. The detailed implementation process of the field-guided museum visit was described elsewhere [24]. In brief, it includes four steps: observation (5 min), interpretation (10 min), reflection (5 min) and communication (10 min). Participants were encouraged to identify objective visual findings without judgment and draw conclusions about the meaning of the art pieces.

In the second session (exposure to clinical images with debriefing), participants were asked to replicate the approach they learned in the first session to observe four clinical images with specific disease processes or visible clinical symptoms (diabetic foot, ascites, dyspnea and debilitation) and to discuss plausible nursing diagnoses. These images were taken by nurses" in different wards according to the following requirements: 1) one to three people (including patients, nurses, and physicians, etc.) in the image to ensure richness of the visual information without getting too jumbled, 2) avoiding posing to promote authenticity, and 3) with permissions to be used in teaching. The images used for intervention got the consensus of at least five clinical or nursing professors. This session was guided by a nursing professor and lasted approximately 1 h (each clinical image for 15 min). For the fidelity of this intervention, the museum guides strictly adhered to the teaching manual with a trained research assistant in each group to remind them of any missed points or excessive information.

### Data collection

#### Instruments

***Demographics and Art-related Experience*** were collected via a self-administered questionnaire. Demographics included age, gender, types of masters, and work experience. Art-related experience included duration of exposure to formal and general art education.

***Observational and Diagnostic Skills*** were measured with a self-administered “clinical image test” which included an Observation Subtest and a Diagnosis Subtest. The two subtests involved four clinical images (none of the images used in the test were discussed before). The clinical images were different at baseline and 3-month follow-up, but comparable images (subject, genre, and complexity) were selected from a large group of images by experienced nursing professors.

The “clinical image tests” are reliable and valid tool [25], and are the mainstream way of evaluating observational and diagnostic skills across countries [9]. We recorded its test-retest reliability and inter-rater reliability of the “clinical image tests” in this study, and evaluated the content validity before using it.

As for test-retest reliability, 12 participants were invited to take the “clinical image tests” 2 weeks (±3 days) before baseline and thus the test-retest reliability can be calculated by combining their baseline data. In this study, the test-retest reliability is r = 0.873. As for inter-rater reliability, two graders were invited to score the “clinical image tests”. We measured the correlation of the scores rated by two graders using Pearson correlation. In this study, we obtained excellent inter-rater reliabilities (r = 0.84) and confirmed the homogeneity of all the images used in this study.

As for content validity, we invited six experienced nursing professors to assess clinical images one by one. These clinical images were from the image pool which taken by nurses in different wards. Only images which earned the approval of at least five experienced nursing professors were selected in the “clinical image test”. After that, the same six nursing professors were asked to comment on the content validity of the selected images using Context Validity Index (“1” = “not relevant”; “2” = “somewhat relevant”; “3” = “quite relevant”; “4” = “highly relevant”). All images in the “clinical image test” were determined to be highly relevant (4/4 points) to evaluation of observational or diagnostic skills by at least five of six nursing professors and thus the “clinical image tests” were regarded as having an acceptable content validity.

Participants were given 40 min to complete the “clinical image test” and reported their free-text observations and diagnoses as fully and accurately as they could. Two trained graders (nursing masters, who masked to the group) independently scored the test by using a predetermined grading list. The average score of the two graders was taken as the final score. To obtain the grading list, five nursing professors independently recorded their observations for each image and then gathered together to reach a consensus regarding the grading list before the trial. A “point” was assigned for each objective observation if they matched the predetermined grading list. The proportion of the obtained points to the total possible points (the pool of observations for this image based on discussion of five nursing professors) were calculated. Higher proportions indicate better observational skills. The scoring method also applies to the evaluation of diagnostic skills by the “clinical image tests”.

***The Learning Satisfaction with the Culturally-tailored Training Rated by Students*** was measured with a self-administrated satisfaction questionnaire (30 items) on a 5-point Likert-type scale from rating 1 = very dissatisfied to 5 = very satisfied. It involved Chinese oil paintings, museum, staff, and training content. The total satisfaction score was defined as the mean of all item scores. The Cronbach’s alpha was .96 in this study.

***The Teaching Satisfaction with the Culturally-tailored Training Rated by Staff Members*** was measured using two self-designed questions on “whether you will recommend this training to your students?” and “what is your favorite part in this training?”

#### Procedures

Outcome variables included observational skills, diagnostic skills, and learning satisfaction with the culturally-tailored visual art training rated by students and teaching satisfaction rated by staff. All the variables were measured by self-administered questionnaires. Data on observational and diagnostic skills were collected at baseline and 3-month follow-up by two trained research assistants who did not know the group assignment. Data on learning and teaching satisfaction were collected right after the intervention. Participants were invited to complete these questionnaires in a quiet classroom. Research assistants were available to answer questions and check each questionnaire to avoid unintentional missing items or pages.

## Data analysis

All data were double entered in a database and checked for accuracy. All analyses were done using SPSS (version 23.0; SPSS Inc., Chicago, IL). A *p*-value < 0.05 was considered statistically significant. Demographics between the intervention and control groups were compared with two independent samples t-test and Chi square tests. Changes in scores of observational and diagnostic skills were analyzed using independent sample t-tests according to the difference in differences method. Descriptive statistics were computed to describe the learning and teaching satisfaction with the culturally-tailored visual art training.

## Results

**Fig. 1:**
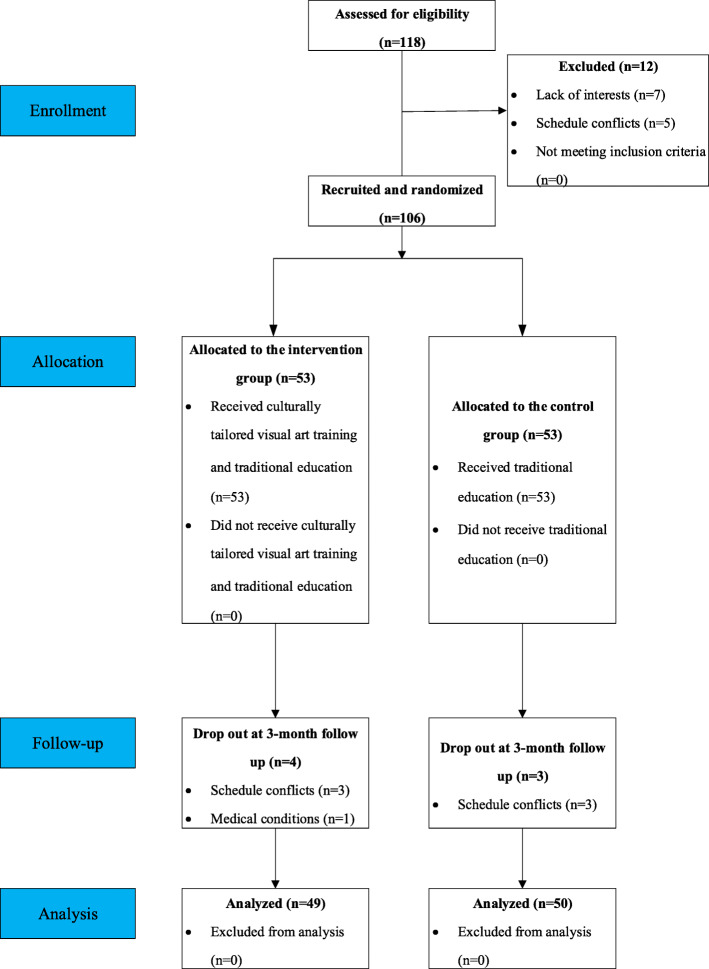
The CONSORT participant flowchart

Of the 118 students who were invited to participate in the study, 12 students (10.2%) declined participation due to lack of interest or schedule conflicts, etc. Attrition at 3-month follow-up was 6.7% (7/106), with the rest of 99 (49 in the intervention group and 50 in the control group) analyzed. There was no significant difference in the demographics and art-related experience between the seven participants who dropped out and the 99 participants who completed this study (*p* > .05). Figure 1 shows the CONSORT participant flow chart.

### Comparison of demographics and art-related experience at baseline between intervention and control students

The mean age of participants was 26.2 (*SD* = 4.1) years, with 26.0 (*SD* = 4.7) years for the intervention group and 26.3 (*SD* = 3.3) years for the control group. A total of 94% of the participants were female, and 41% were full-time nursing students. The work experience of participants was 3.4 (*SD* = 3.3) years on average. A total of 57% of the participants reported that they had received general art education for 6 years or fewer, and 67% never received professional art education. There were no significant differences between the two groups at baseline for demographic and art-related experience (*p* > .05), as reported in Table 1.

**Table 1:** Comparison of demographics and art-related experience at baseline between intervention and control students

Characteristics	Intervention (*n* = 53)	Control (*n* = 51)	χ^2^/*t*	*p*-value
Age (years), mean (*SD*)	26.0 (4.7)	26.3 (3.3)	0.34	.733
Female, *n* (%)	49 (93)	49 (96)	0.14	.678
Full-time nursing students, *n* (%)	25 (47)	18 (35)	1.51	.219
Have been a nurse for (years), mean (*SD*)	3.0 (3.6)	3.9 (3.4)	1.18	.239
Received general art education (years), *n* (%)			0.59	.444
≤ 6	32 (60)	27 (53)		
> 6	21 (40)	24 (47)		
Received formal art education (years), *n* (%)			0.95	.331
Yes	15 (28)	19 (37)		
No	38 (72)	32 (63)		

*Note. SD* Standard Deviation

### The 3-month efficacy of culturally-tailored visual art training on observational and diagnostic skills

As shown in Table 2, the proportion (raw obtained scores/total possible scores, range 0–100%) of objective observations made by intervention students (mean change + 9.84%, *SD* 11.29%) significantly increased compared with that of control students (mean change − 0.72%, *SD* 7.44%, *p* < .001). A trend towards the improvement of diagnostic skills was indicated with increment of 2.92 points (SD 13.71) of the intervention group vs. 0.39 (SD 10.89) of the control group during 3-month study period, though there was no statistical difference between the two groups (*p* > 0.05).

**Table 2:** The 3-month efficacy of culturally-tailored visual art training on observational and diagnostic skills

		Intervention group (*n* = 49) Mean (SD)	Control group (*n* = 50) Mean (SD)	t	*p*-value
Observational skills	Baseline	17.39 (7.82)	16.17 (8.92)		
	3-month follow-up	27.15 (10.90)	15.45 (6.91)		
	Change in means	9.84 (11.29)	−0.72 (7.44)	5.615	<.001
Diagnostic skills	Baseline	23.13 (8.18)	21.96 (7.42)		
	3-month follow-up	26.06 (12.66)	22.35 (10.21)		
	Change in means	2.92 (13.71)	0.39 (10.89)	1.041	0.301

*Note. SD* Standard Deviation

### Satisfaction with culturally-tailored visual art training

The mean score of satisfaction rated by the intervention group was 4.5 out of 5 points (range from 3.3 to 5.0) on a 5-point Likert scale. The top two items with the highest score in the satisfaction questionnaire were the selected Chinese oil paintings with all participants (*n* = 53) satisfied or very satisfied, and degree of involvement in the training with over 90% of participants (*n* = 48) satisfied with their active involvement in the training. What students were most dissatisfied with was the distance to the museum, with 35% (*n* = 18) participants choosing “dissatisfied” or “very dissatisfied”. Over 20% of participants (*n* ≥ 11) were dissatisfied or very dissatisfied with the length of visual art training and its practical utility. All of the staff members indicated that they would recommend this culturally-tailored visual art training to their students and half of the staff (*n* = 4) said interpretation process of Chinese oil paintings was their favorite part.

## Discussion

To the best of our knowledge, we are the first institution to introduce visual art training to Mainland China and adapt the training for the Chinese culture among nursing students. We confirmed culturally-tailored visual art training can improve observational skills of Chinese nursing students in master program. The Chinese oil paintings with rich Chinese characteristics used in the training are appropriate to be selected as the main teaching resources, which was responsive to the Chinese cultural context and suitable for Chinese nursing students.

A systematic cultural adaptation was conducted based on a multi-phase mixed method study design guided by a heuristic framework [20], rather than delivering a fixed intervention. Kumpfer et al. [26] indicated that cultural adaptations are imperative when engagement is below standards that were established in applications of foundation researches, as it might affect the core components of the training that account for its efficacy. We used a qualitative approach to integrate the concerns of relevant stakeholders, who assess characteristics and preferences of Chinese nursing students (e.g. tend to be shier) and identify ideas that have the best chances to enhance the student involvement to better translate the visual art training in Mainland China.

The culturally-tailored visual art training could significantly improve observational skills 3 months after the training, which is consistent with the findings of prior research suggesting that observational skills could be enhanced with focused viewing, describing, and interpreting of art pieces and debriefing clinical images [27]. Based on the 3-month follow-up efficacy, we confirmed that visual art training is effective for a longer follow-up period in improving observational skills.

In our study, there was an exciting trend towards the improvement of diagnostic skills during 3-month study period. According to a guideline of visual art, diagnostic skills are hard to improve in the short term without a well-developed systematic knowledge of nursing, although the improvement of observational skills creates the conditions for the improvement of diagnostic skills [28]. Nursing postgraduate education in China is still in its infancy [29]. Many components of its curriculum (for instance, objectives, requirements, and evaluation) are not appropriate for Chinese nursing students [30]. We believe diagnostic skills can be improved in the future as the nursing knowledge of students improves.

Given the satisfaction survey of culturally-tailored visual art training we carried out among students and the staff, it is clear that the cultural adaptation of visual art training was successfully completed in China as the pre-identified cultural barriers were overcome. It also shows that there is a need to increase the amount or duration of visual art training. A positive dose-response relationship was also demonstrated between the number of visual art sessions attended and the improvement of observational skills accordingly [25]. Some participants also indicated that the museum is too far, which is the challenge that most developing countries have faced—scarce museum resources. Although we got access to the museum, seeking an alternative to museum visits is a future research direction.

Compared with existing studies, the strengths of our study included a well-crafted culturally-tailored visual art training based on stakeholder’s comments, subject randomization, masking of graders, and a predetermined grading list used to standardize grading.

### Limitations

This study has several limitations. First, clinical outcomes were not measured. This means there is no direct evidence that these skills can be transformed into improved patient care. We used clinical images as reasonable surrogates in this study, which are the main tool for testing observational and diagnostic skills used in the literature, but it would be worthwhile to evaluate the students using actual (or standardized) patients and add self-assessment in future studies. Second, the comparability of the images at baseline versus 3-month follow-up might be questioned, although we created a measurement standard. Third, the improvement of skills is a slow process, usually more than 3 months, and thus evaluation of observational and diagnostic skills after 3 months is insufficient.

### Implications

There are several important implications for both of teaching practice and research. First, the systematic cultural adaption process of an innovative teaching approach modified for Chinese culture could provide an example to disseminate the evidence-based program to other countries with different cultures. Second, there is considerable evidence that excellent observational skills can provide information critical to the accuracy of diagnoses, which is the ultimate goal of the visual art training applied in nursing education. More studies focused on diagnostic skills are needed, and how to translate observational skills into diagnostic skills remains an open question. In addition, future research should consider a longer time frame in evaluation of observation and diagnostic skills and use a more objective and standardized instrument for outcomes assessment.

## Conclusions

The systematic cultural adaption process of the visual art training modified for Chinese culture provides a reference for other places with different cultures to localize this training and make it available to local nursing students. Culturally-tailored visual art training via Chinese oil paintings is feasible and acceptable for Chinese nursing students during a longer follow-up period. Further studies for longer period are needed on diagnostic skills and on how skills gained in visual art training translate to clinical practice.

## Data Availability

The datasets used and analyzed during the current study are available from the corresponding author on reasonable request.

## Appendix

**Table 3:** The main comments concerning visual art training in the pilot

No.1 participant	➢ After the visual art training, I realized I missed so many key points in my previous observation. ➢ The observation procedures I learnt from the training really help me to make nursing diagnosis.
No.2 participant	➢ I think it is interesting and I want to take an active part. ➢ It was hard for me to initiatively interpret these Western art pieces without cues from the teacher.
No.3 participant	➢ I think most Western art pieces had intricate historical backgrounds and mysterious religious color for me. ➢ Agree with No.2 participant.
No.4 participant	➢ I don’t know how to generate a plausible explanation based on this unfamiliar information. ➢ I don’t want to be selected as a spokesman and get involved in discussion, because when I say something wrong, I will feel embarrassed.
No.5 participant	➢ It was an exciting and novel experience. ➢ I believe our observational and diagnostic skills will be improved with the help of visual art training.
No.6 participant	➢ My favorite part is observation and interpretation of clinical images. I think it is more clinically relevant to my later professional life. ➢ Agree with No.4 participant.
No.7 participant	➢ I was satisfied with visual art training. ➢ Agree with No.2 participant.
No.1 museum guide	➢ Students seems to have difficulty in concentrate on observation. ➢ In most cases, we have to propose our ideas and students just follow us instead of proposing their own thinking from a different perspective.
No.2 museum guide	➢ I think Chinese oil paintings are easier for Chinese students to understand compared with Western art pieces. ➢ Replacing the Western art pieces with Chinese oil paintings may greatly help us delivering this program.
No.3 museum guide	➢ Why do not we deliver the program in an art museum? Compared with simulated museums, a real art museum has more paintings and better learning atmosphere to inspire students get more involved in the training.
No.4 museum guide	➢ It is important for us to encourage students to overcome their shy nature. ➢ Agree with the clinical professor.
Art Professor	➢ There was an unconscious shift from student- to teacher-led training during the implementation process, which was not what we expected. ➢ This might be due to shy nature of Chinese students and obscure Western art pieces. We should work on this.
No.1 nursing professor	➢ Students need more time to share ideas and generate multiple interpretations via group discussions. ➢ Maybe we can prolong the session for interpretation and communication to help improve student involvement.
No.2 nursing professor	➢ I suggest the selected paintings should have rich visual detail, and potential for ambiguity of interpretation without predetermined correct answers. ➢ Agree with No.1 museum guide
Clinical professor	➢ We need to encourage students to guess and interpret paintings based on their observation as boldly as possible. ➢ We will correct them only when their interpretations were illogical or showed idiosyncratic personal bias.
